# AM I FRAIL, LOVE? YES, I SUPPOSE I AM: WHAT 10 OLDER PEOPLE CAN TELL US ABOUT LIVING WITH FRAILTY

**DOI:** 10.1093/geroni/igz038.1080

**Published:** 2019-11-08

**Authors:** Oliver M Todd, Andrew Clegg, Mary Godfrey

**Affiliations:** University of Leeds, Bradford, Yorkshire, United Kingdom

## Abstract

We sought to explore what matters in later life with frailty from an older persons perspective. Between March and May 2018, we recruited ten people, purposively sampled from the CARE75+ ageing cohort study. Interviews took place at the participant’s own home in two sittings, each 45 minutes long. Interviews were semi-structured, used narrative techniques based on a topic guide developed with a patient representative. We used systematic analysis of the narrative experience to identify meaning in the context of an individual’s time, space and history. Participants had a mean age of 84 years (range 77 to 93), half were women, and three were interviewed with their care-givers. All had moderate or severe frailty: mean frailty index 0.36 (range 0.25 to 0.47); mean Fried score 4 (range 3 -5). Half knew hunger as children; most grew up in large families and left school early; two survived TB in early life; all lived through or were affected by war. The term frailty was: never voluntarily used; described negatively and in value laden terms; seen better in others than themselves. Decision making was best delegated to doctors who knew you and your family over time. Narratives focused on health events of a spouse; symptoms featured more than diagnoses. Survivorship, reciprocity and community were sustaining values. To engage elders in shared decision making we learnt to consider influences of cohort, of people closest to them; and to describe rather than declare someone to be frail, in terms that are real to them.

